# Seasonal Variation in Human Gut Microbiome Composition

**DOI:** 10.1371/journal.pone.0090731

**Published:** 2014-03-11

**Authors:** Emily R. Davenport, Orna Mizrahi-Man, Katelyn Michelini, Luis B. Barreiro, Carole Ober, Yoav Gilad

**Affiliations:** Department of Human Genetics, University of Chicago, Chicago, Illinois, United States of America; Institut Pasteur, France

## Abstract

The composition of the human gut microbiome is influenced by many environmental factors. Diet is thought to be one of the most important determinants, though we have limited understanding of the extent to which dietary fluctuations alter variation in the gut microbiome between individuals. In this study, we examined variation in gut microbiome composition between winter and summer over the course of one year in 60 members of a founder population, the Hutterites. Because of their communal lifestyle, Hutterite diets are similar across individuals and remarkably stable throughout the year, with the exception that fresh produce is primarily served during the summer and autumn months. Our data indicate that despite overall gut microbiome stability within individuals over time, there are consistent and significant population-wide shifts in microbiome composition across seasons. We found seasonal differences in both (*i*) the abundance of particular taxa (false discovery rate <0.05), including highly abundant phyla Bacteroidetes and Firmicutes, and (*ii*) overall gut microbiome diversity (by Shannon diversity; *P* = 0.001). It is likely that the dietary fluctuations between seasons with respect to produce availability explain, at least in part, these differences in microbiome composition. For example, high levels of produce containing complex carbohydrates consumed during the summer months might explain increased abundance of Bacteroidetes, which contain complex carbohydrate digesters, and decreased levels of Actinobacteria, which have been negatively correlated to fiber content in food questionnaires. Our observations demonstrate the plastic nature of the human gut microbiome in response to variation in diet.

## Introduction

The human gut microbiome varies greatly in content across individuals, both within and between populations [1], [2], and is believed to be influenced by many factors such as method of delivery at birth [3], [4], antibiotic usage [5], [6], and disease status [7]–[9]. Nonetheless, diet is intuitively considered one of the most important determinants of the gut microbiome composition both in infants [10], [11] and in adults [12]–[14].

Despite this clear intuition, our understanding of how diet affects microbial abundance and to what extent is still limited. More generally, we do not yet know how stable or plastic the gut microbiome is in humans in response to diet. Work in humanized, gnotobiotic mouse models has provided insight into the effects of variation in diet on microbiome composition by mimicking human diets in mice, and has revealed the disease-inducing potential and metabolic capabilities of the microbiome [15]–[18]. Yet, for a number of reasons, it is difficult to infer the extent of dietary effects on the human microbiome from studies in model organisms. First, mouse models usually derive their “human” microbiome from a small number of sources and thus are not typically reflective of the variation found in a natural human population. Second, the low genetic diversity, strict diets, and extremely homogenous environments of laboratory mice throughout the course of experimentation (usually an advantage of working with model systems) are not representative of typical environmental and dietary exposures in humans. As a result, surveys of the human microbiome both within and across populations are necessary to complement results from studies in mouse.

Human studies, however, are challenging because it is impractical to control the subject's environment for long periods of time and hence difficult to test the effect of only one variable, such as diet. Several studies performed in humans have attributed alterations in the gut microbiome to diet [19]–[21], but in many cases other variables known to affect the microbiome were not accounted for or were confounded with diet. For instance, inter-population comparisons, such as between Italian and Burkino Fasan children or between Americans, Malawians, and Amerindians, measured gut microbiome differences that are likely associated with differences in climates, genetic background, access to medical care, sanitary practices, and pathogen exposure across populations, in addition to differences in diet [2], [22], [23]. One of the most well controlled within-population studies that examined dietary affects on gut microbiome was done in the elderly; however, diet was still confounded with residency status (living in the community or long-term stay facility; [24]. Recently, the gut microbiomes were examined in individuals who were either put on a regimen of strictly plant-based diets or animal diets for several days, preceded by a baseline measurement and followed by a washout period [25]. There were significant shifts in microbiome composition and function during these interventions, however, it is unclear how generalizable these results are given that the diets were very strict and the study included only 11 individuals.

In an attempt to circumvent concerns regarding confounding variables, other studies controlled environmental influences through dietary interventions or short, sequestered hospitals stays [12], [26]–[29]. Although these approaches minimize environmental variation for the duration of the study (typically no longer than two weeks for sequestration or several months for dietary interventions), lifetime exposures prior to intervention may still have lasting effects through the study.

Perhaps the strongest evidence that natural dietary differences between individuals are directly associated with variation in adult microbial composition was provided by a study that used food questionnaires to examine how the microbial content of the gut was associated with the intake of specific nutrients [12]. Although the major conclusion was that long-term dietary habits were correlated with changes in enterotypes [30], the three divisions of individuals based on their gut microbiota, specific significant associations between nutrient intake and variation in the abundance of individual taxa were also observed. For instance, the abundance of certain microbial taxa showed inverse associations with fat intake and positive associations with plant-derived nutrients. This study provided one of the first detailed insights into how specific bacteria might interact with components of the diet.

The optimal study of the effects of diet on the gut microbiome would include proper long-term control of environmental variation on the one hand, and direct manipulation of diet on the other hand. While it is probably impossible to design an optimal study in humans, we found that we can sidestep many of the obvious limitations by studying the gut microbiome in an isolated, communal-living population: The Hutterites. The Hutterites in North America descended from a small, founder population from Europe who emigrated between 1874 and 1879, and have grown to a community with >30,000 members living on communal farms across the northern U.S. plains states and western provinces of Canada [31]. This population presents several key advantages for microbiome research. First, unlike some other isolated groups, the Hutterites use technology (exposed to medical care, electricity, cars, etc.), making them a reasonable proxy for populations from developed countries. In addition, the genetic variation between Hutterite individuals is lower compared to the general American or European population because they descended from a small number of founders and gene flow into the population has been limited. Also, because of their residential stability and limited exposure to the outside, environmental exposures are less heterogeneous both within individuals over time and across individuals at any one time.

Most importantly in the context of a gut microbiome study, the Hutterites live on communal farms (called colonies). Their three main meals are prepared and eaten in a colony dining room, using traditional recipes that have been relatively stable over time and between colonies. The main source of variation in their diet is the seasonal availability of fresh produce, which is primarily grown on the colonies. Specifically, during the summer and autumn months a variety of fresh fruits and vegetables are grown, consumed, and prepared for consumption throughout the year. During the winter months, produce consumption primary consists of the fruits and vegetables that were preserved, canned, or frozen during the summer months.

In this study, we examined the temporal stability of the human gut microbiome in the Hutterites during both summer and winter months of one year. The study design, as well as the characteristics of Hutterite lifestyle, gives us a unique opportunity to study the gut microbiome over time in a natural population with minimal environmental and genetic variation, in order to assess how seasonal differences in diet shape the gut microbiome.

## Results

In order to characterize temporal variation in the gut microbiome of the Hutterites, we sampled stool from the same 60 individuals in the winter and summer months of one year. The individuals included in the study had not taken antibiotics for 6 months prior to either sampling period. From each sample, we amplified the V4 hypervariable region of the 16S rRNA gene and sequenced the sample using a HiSeq 2000 (see methods). We used two separate DNA extractions from each stool sample as technical replicates.

**Figure 1:**
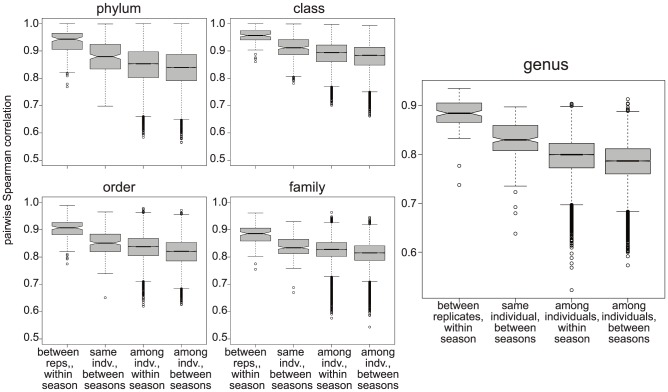
Correlation of microbiome composition between replicates, individuals, and over time. Boxplots of pairwise Spearman correlation (y-axis) of bacterial abundance is shown for data at each taxonomic level (x-axis) across different classification levels. ‘Replicates’ refer to technical replicates derived from separate DNA extractions and library preparations from the same sample.

We were able to characterize both common and rare taxa in each sample by using 2 million sequence reads per technical replicate (4 million reads per sample; see Figure S1 and Table S1). We observed low technical variability associated with the extraction and library preparation protocols (Figure 1), and thus combined reads across replicates for all subsequent analyses.

### Assessing the temporal stability of the microbiome

To examine the similarity of the microbiome composition across individuals and within individuals over time, we examined pairwise Spearman correlation of abundance of all taxa identified at each taxonomic level (Figure 1). Our data indicate that the microbiome composition varies more across individuals (genus level mean r^2^ = 0.795) than within individuals over time (genus level mean r^2^ = 0.876). We did not find higher variation in the microbiome of individuals residing in different households compared with individuals sharing the same household even considering relatedness (Figure S2). This observation indicates that, regardless of household, Hutterites share much of their environment.

**Figure 2:**
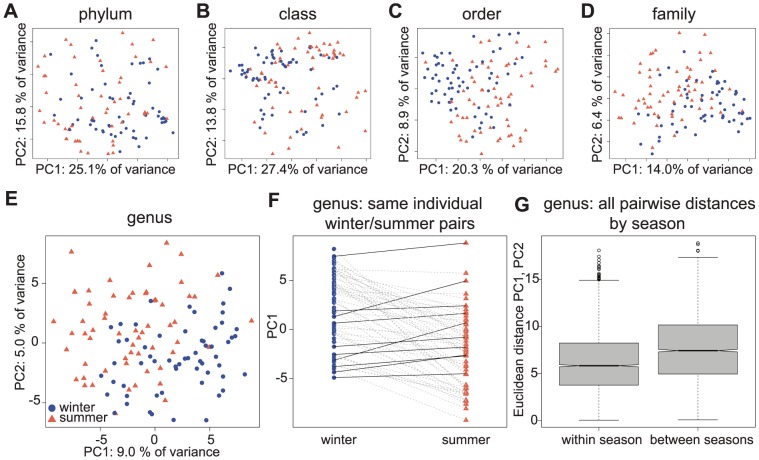
Consistent temporal shifts observed in microbiome composition. Principal components analysis (PCA) of data obtained from samples collected in the summer (red triangle) and winter (blue circle), at the phylum (A), class (B), order (C), family (D), and genus (E) level across samples. F) Individual shifts in microbiome composition along PC1 (y-axis). Data from the same individual across seasons is connected by a line (gray lines represent individuals showing seasonal trend, black lines highlight individuals showing the opposite trend). G) The average pairwise distance along the PC1/PC2 plane is shorter where points are from the same season than points between seasons (t-test *P* = 3.38×10^−78^), supporting seasonal clustering along the first two principle components.

We observed a clear seasonal effect on microbiome composition. The data cluster by season through principal components analysis (PCA), especially at the lower taxonomic ranks, albeit with a great deal of overlap (Figure 2 and Figure S3). The consistent shift in the summer and winter clusters across the first principal component (for nearly all samples; Figure 2f) demonstrates that there are consistent composition differences across the two seasons, rather than stochastic variation in abundances of particular taxa over time. It is important to note that individuals were sampled on six separate colonies and the seasonal trends are consistent across colonies.

Significance of the clustering was examined in two ways (Figure 2g). First, the averages of all-pairwise Euclidean distances along the PC1/PC2 plane were compared between all pairs of samples within season to all pairs of samples between seasons. The distance between pairs of data points along the plane of the first two principle components within season is significantly shorter than between seasons (t-test *P*<0.001), indicating that samples cluster by season. Second, we performed a nearest neighbor test as described by da Silva et. al [32] and found that the three nearest neighbors to any given point along the PC1/PC2 plane are significantly more likely to be from the same season (*P*<0.001).

To robustly identify particular bacteria that are differentially abundant across seasons, we performed several tests for differential abundance. Two versions of a metastats test are reported: one incorporates a paired t-test and the second uses a paired Wilcoxon rank sum test [33]. In addition, due to concerns regarding randomly subsampling reads in microbiome studies, two versions of metagenomeSeq differential abundance tests are reported: one uses subsampled reads and the other uses all of the reads sequenced per sample and includes a parameter to account for library size [34].

**Figure 3:**
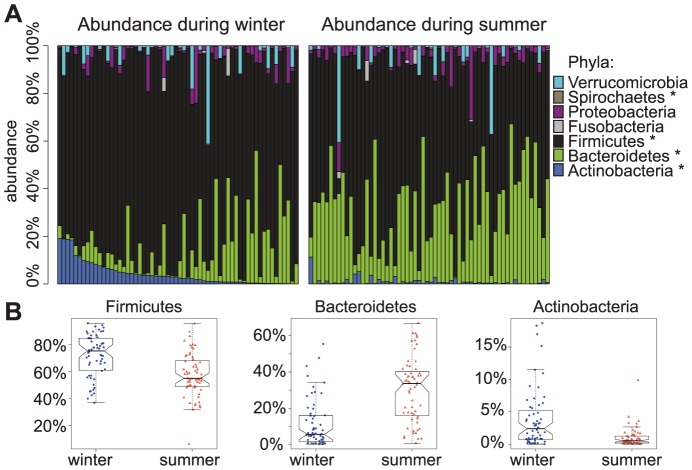
Phylum level taxa abundances differ by season. A) Abundance (y-axis) of the 25 bacterial phyla that were detected (only the most common bacteria are indicated in the legend) by individual (x-axis). Individual's order along the x-axis is identical in both panels. Phyla that are significantly differentially abundant between seasons (FDR <0.05) are indicated by an asterisk. B) Examples of three common phyla whose abundances (y-axis) are significantly different across seasons (x-axis): Firmicutes (q-value<0.002), Bacteroidetes (q-value<0.002), and Actinobacteria (q-value<0.002).

The altered metastats test (incorporating a paired t-test) for each taxon at all ranks resulted in the smallest number of tests in which the null was rejected (Tables S2, S3, S4, S5; Figure S4). At a 5% false discover rate (FDR), we observed consistent, significant abundance differences between seasons across all levels of the taxonomy examined (phylum: 10/25, class: 15/35, order: 32/67, family: 45/144, genus: 94/316; Table S2) and among common and rare bacteria alike (Figure 3a). For example, we found abundance differences between seasons in some of the most rare bacterial phyla, such as Chloroflexi and Gemmatimonadetes, as well as some of the most common bacterial phyla, such as Bacteroidetes, Firmicutes, and Actinobacteria (Figure 3b).

Following previous work [12], [30], [35], we also examined whether enterotypes can be distinguished in our data. We do not find strong evidence for enterotypes in either season in this population, either when seasons are analyzed together (silhouette score <0.5, indicating low support for the best number of clusters; [36]), or separately, where we observe different numbers of clusters depending on season (winter k = 3, summer k = 4, both associated with silhouette scores <0.5; see methods and Figure S5).

### Diversity varies by season and age, but not by sex

**Figure 4:**
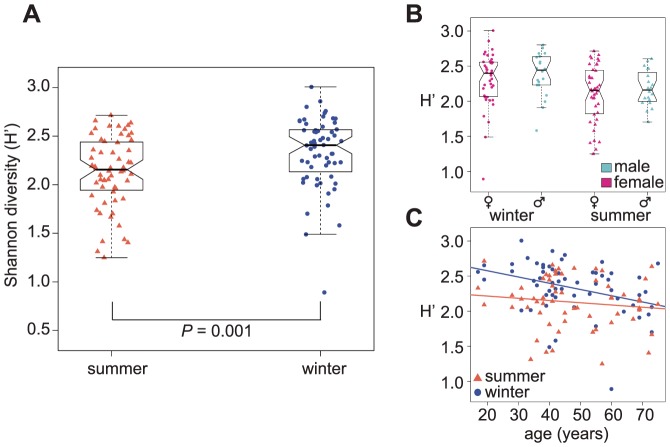
Diversity between seasons, sexes, and age. A) A significant difference (paired t-test *P*<0.002) in Shannon diversity (H′, y-axis), between season (x-axis). B) H′ between sexes is not significantly different in either winter (t-test *P* = 0.46) or summer (*P* = 0.38). C) Diversity significantly decreases with age based on the data collected from winter samples (r^2^ = 0.1267, *P*<0.006), but not based on the data collected from the summer samples (r^2^ = 0.01653, *P* = 0.33).

We examined the diversity of the gut microbiome within season. We observed significantly higher diversity during the winter sampling period than the summer (paired t-test using Shannon diversity metric (H), *P* = 0.001; Figure 4a). Diversity was not significantly different between sexes in either season (*P* winter = 0.45 and *P* summer = 0.38; Figure 4b). There was a slight inverse trend of gut microbiome diversity with age in the winter (*r^2^* = 0.1267, *P*<0.006) but no discernable relationship in summer (*r^2^* = 0.01653, *P* = 0.33; Figure 4c). In addition, we examined each of these comparisons using richness and evenness, and observed similar trends (Figure S6).

### Produce consumption varies by season

In order to formally assess variation in the Hutterite diet between seasons, we asked the donors who provided stool samples to complete daily food questionnaires during 7 consecutive days in each season. We were able to collect completed questionnaires from 31 and 28 individuals in the winter and summer, respectively. These questionnaires were modeled based on semi-quantitative Food Frequency Questionnaires [37], altered to ask more general trends (for example, we asked “how many times did you eat fresh fruit today?” rather than which fruit was eaten; Table S6). In addition, we combined specific questions into broad categories of food (for example, produce, dairy, carbohydrates, etc.) to identify seasonal trends.

**Figure 5:**
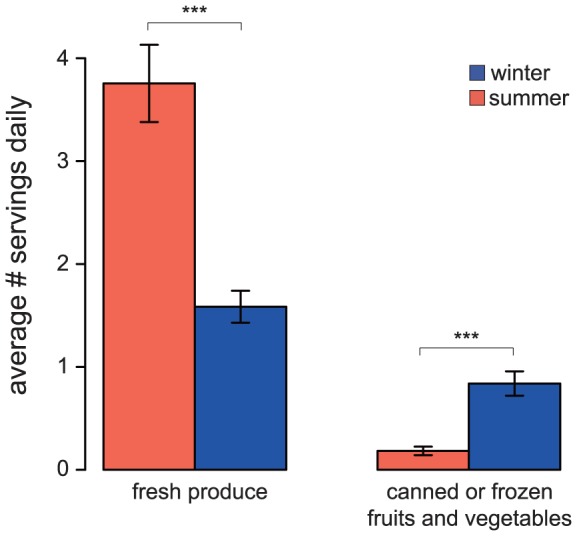
Produce consumption varies by season. In summer, when fruits and vegetables are grown in colony gardens, consumption of fresh produce is higher than in winter. Conversely, when fresh produce is not as available, a higher proportion of canned or frozen fruits and vegetables are eaten. Bars indicate standard error measurements. *** P<0.001 (t-test, adjusted for multiple tests using Benjamin Hochberg correction). See Table S6 for a list of all tests performed.

Based on these food questionnaires, Hutterite diets differ between seasons primarily with respect to the amount of produce consumed (Table S6). Specifically, we observed seasonal differences in several individual produce categories, including fresh vegetables (adjusted *P*<0.0002), frozen or canned vegetables (adjusted *P*<0.0003), corn (adjusted *P*<0.02), and frozen or canned fruit (adjusted *P*<0.02). Considering broader categories, our data indicate a marked seasonal difference in the consumption of fresh produce (fresh fruit and fresh vegetables combined, adjusted *P*<0.0002), the consumption of frozen or canned fruits and vegetables (adjusted *P*<0.0002), and in the amount of produce eaten in general (adjusted *P*<0.02, Figures 5 and Table S6 and Figure S7).

## Discussion

We characterized the gut microbiome of individuals from a unique population, the Hutterites, whose communal lifestyle minimizes environmental variation across individuals (compared to other sampling schemes in humans). The microbiome of the same individuals was examined during summer and winter months, when the Hutterite diets differ primarily with respect to the proportion of fresh produce they consume. Our goal was to assess the temporal stability of the microbiome and the role that dietary perturbations play in shaping the composition of the microbiota. Similar to other recent studies, we observed a high degree of temporal stability in members of the microbiome within an individual over time [38]. However, there are significant shifts in bacterial abundance and diversity between seasons in this population.

### Seasonal shift in diet as a likely explanation

Many physical environmental factors (which we did not document) potentially differed by season in this population, including the amount of sun exposure, temperature, humidity, and the relative amount of time spent outdoors. It is likely that many of these seemingly unrelated factors influence the human gut microbiome in ways that are not yet understood. For instance, seasonal differences in day length have been shown to influence the composition of the gut microbiome in rodents [39]. In addition, it has been well documented that there is seasonal variation in pathogen exposure and immune response for various well known pathogens, therefore, some variation across seasons is to be expected [40]. That being said, we observed large-scale temporal differences in the microbiome in this population that span multiple phylotypes of bacteria and were consistent across many geographically isolated sites.

Although the environmental factors listed above potentially influence the gut microbiome to some extent, a more likely explanation for the large scale differences we observe could be due to diet. Differences in diet potentially alter the microbiome by exposing individuals to different microbes [41] or by changing the gut nutrient content, thereby providing a better niche for certain bacteria. In addition, nutrition itself is known to modulate an immune response [42], which potentially influences microbiome composition. Although many of our observations are consistent with diet influencing the microbiome in these individuals, further testing must be done to prove causality.

Consistent with the notion that diet drives seasonal differences in the microbiome, we observed higher relative abundance of the phylum Bacteroidetes, one of the most common and prevalent groups of gut bacteria, during the summer compared to the winter months (mean summer abundance = 30.3%, mean winter abundance = 11.2%, q-value<0.002). The Bacteroidetes clade is known to contain specialists in degrading high molecular weight organic material, such as plant cell walls and complex carbohydrates, and their genomes are known to contain large numbers of CAZymes, or carbohydrate-active enzymes [43], [44]. The increase in the relative abundance of this phylum in the summer is consistent with our observation that the individual diet of the Hutterites included a greater consumption of fruits and vegetables in summer compared to winter (adjusted *P*<0.2). Indeed, metagenomic prediction from 16S using PICRUSt [45] reveals a significant increase in KO enzymes that are categorized under the “Glycan Biosynthesis and Degradation” pathways in summer as compared to winter (*P = *1.08×10^−6^, paired Wilcoxon rank sum test, Figure S8, Table S7). The increased intake in plant matter potentially gives Bacteroidetes a nutritional advantage and allows this clade to bloom during the summer months.

This inference is also consistent with other previous observations. For example, a study that compared the gut microbiomes of children from Italy and Burkino Faso reported a higher level of Bacteroidetes in the African children than the Italians, perhaps due to the high levels of complex starches in the African diet [22]. In addition, the observation of a decrease in the ratio of Bacteroidetes to Firmicutes in lean compared to obese individuals [7], [46], [47] may be at least partially due to differences in diet. This has previously been indicated by a study in a mouse model of obesity [48]. Specifically, Bacteroidetes abundance was reduced when animals were placed on a high fat diet, regardless of their genetic susceptibility to obesity.

We also found that Actinobacteria abundance increased in the winter compared to the summer months (mean winter abundance = 3.84%, mean summer abundance = 0.10%, q-value<0.002). Previously, Wu et al. observed a positive correlation of Actinobacteria abundance with the amount of fat consumed and a negative correlation with the amount of fiber consumed. These results are consistent with the observed seasonal variation in produce consumption (increased produce consumption in summer implies a high amount of fiber consumed in this season). These observations provide further support to the notion that dietary differences are potentially responsible for the shift in microbiome composition we observed between seasons. We also found significant seasonal shifts in the abundance of quite a few other types of bacteria (for example, phyla Chloroflexi, Gemmatimonadetes, TM7, etc.). Little is known about many of these bacteria and further research should be done to assess their roles in the gut microbiome and the possible connection to variation in diet. In addition, studies of causality will be essential to prove whether diet is truly responsible for the temporal composition difference we observe.

We note that the seasonal shift in diversity in our data did not match our (perhaps naïve) intuition. We observed decreased gut microbiome diversity in summer compared to winter, though the diet is more varied in summer (with the availability of fresh produce). Our working hypothesis to explain this unexpected result is that certain types of bacteria flourish and outcompete other bacteria when complex starches are available as a nutritional substrate, for instance members of the Bacteroidetes clade. As a result, overall microbiome diversity may be lowered, as many taxa would have lower abundance in summer compared to winter. However, further testing will be needed to provide support to this theory.

### Comparisons to other populations

Comparisons between 16S rRNA studies can be challenging, especially for the gut microbiome, because the high technical variation between studies can potentially mask significant biological differences [49]. With that caveat in mind, we compared the Hutterites to a study that examined the gut microbiome of American, Amerindian, and Malawi individuals using the same sequencing protocol by Yatsunenko et al. [2] and reclassified and analyzed their sequences through our bioinformatics pipeline to reduce technical variability. Perhaps unsurprisingly, we observed smaller scale differences in composition between our winter and summer samples than the shifts previously observed between the gut microbiome of Americans and the Malawians/Amerindians (see methods, Figure S9). Indeed, even if diet is the sole driver of variation across populations in Yatsunenko et. al. it is reasonable to assume that the dietary differences in the Hutterites between seasons are likely much more nuanced (availability of fresh produce differed by season, but otherwise recipes are shared between seasons) compared to the dietary differences across the three populations.

Contrary to the Yatsunenko et. al. study [2] performed across three populations, we did not observe an increase in microbiome diversity with age. We found a slight decrease in diversity with age in the winter samples and no relationship during the summer months. The reasons for the apparent discrepancy between our observations and those of Yatsunenko et. al. are unclear (Figure S9). One possibility is that this trend is highly affected by life style and hence differs by populations.

Finally, we failed to find evidence for enterotypes in the gut microbiome of the Hutterites. We found little support for clustering of the data in either season, and we observed a variable number of clusters depending on whether the data from the two seasons were analyzed together or separately. Moreover, the weakly supported clusters we did observe were not distinguished by the same types of bacteria that were previously used to define the different enterotypes (*Bacteroides*, *Prevotella*, and *Ruminococcus*).

## Conclusions

In this study, we demonstrated that there is a large degree of temporal stability in the composition of the gut microbiome of an individual. We also found significant, population-wide shifts in the microbiome between seasons across multiple independent settlements of our population, which are potentially driven by seasonal dietary differences in the Hutterites. Our results also indicate that population-wide samples taken at one time-point for a study might not capture the entire variation that exists in that population over time.

## Materials and Methods

### Ethics Statement

The protocol was approved by the University of Chicago IRB (protocol 10-416-B). Written informed consent was obtained from all adult participants and the parents of minors. In addition, written assent was obtained from minor participants.

### Sample collection

Participants (> age 16) provided fecal specimens during winter (January/February 2011) and summer (July 2011) months. Sampling was done across 5 different Hutterite colonies, located in South Dakota within 15–20 miles of each other. We collected samples from a total of 124 individuals in winter and 103 in summer. We excluded any individuals from analysis that had taken antibiotics within 6 months prior to sampling, which left 94 individuals in the winter and 92 in the summer (the “full” set). Of the full set of individuals, 60 (40 females and 20 males) provided fecal samples in both seasons. The results of the main paper focus on these 60 individuals; however, for several analyses we present results that consider data from the “full” set of individuals (Figures S3 and S6). The patterns we observe are consistent regardless of the set of individuals used.

Dietary surveys (Table S6) were collected during August 2012 and February 2013, approximately 1 year after the collection of the initial data. Because of this, we analyzed dietary data on a population level to identify seasonal trends and did not relate to the correlation between microbiome composition and diet at the individual level. Of the full set, 41 individuals responded in the winter and 45 individuals responded in the summer. Of the 60 individuals sampled in both seasons, 31 individuals responded in winter and 28 in the summer (24 individuals provided complete questionnaires in both seasons).

### Sample DNA extraction and library preparation

Stool was immediately frozen at −20**°**C after collection, shipped on dry ice, and stored at −80**°**C permanently. Two aliquots of 0.25 g frozen stool were used for DNA extraction per individual per time point, using the Omega Bio-Tek E.Z.N.A Stool DNA Kit (revisions March 2010, Omega Bio-Tek, GA, USA), following provided instructions (Figure S10). DNA concentration and purity were assessed using the Nanodrop 1000 spectrophotometer (Thermo Scientific, IL, USA), and purity was highly similar between the two seasons. The 16S rRNA gene V4 region was amplified and sequenced using the protocol published by Caporaso et al [50] with the following adjustments: 3×50 ng starting template reactions per sample were combined after amplification and samples were quality controlled at the end of library preparation using the Agilent Bioanalyzer DNA 1000 kit (Agilent Technologies, CA, USA).

### Sequencing and classification

Samples were multiplexed 50–80 indices per lane across four flow cells and sequenced using an Illumina HiSeq2000 (CA, USA). Samples were randomized by season and replicate across two flow cells and the remaining two flow cells contained samples from one season each. The 12-cycle index was sequenced first, followed by 102 cycles using the Read 1 sequencing primer. Data were pre-processed using CASAVA 1.8.1 (flow cell 1) and CASAVA 1.7.0 (flow cells 2–4). Reads were quality controlled following the procedure by Caporaso et al. [50] with the following alterations: 1. The beginning of each read was truncated at the point where it incurred two adjacent low-quality base calls within the first 13 base pairs and 2. Low quality was considered Q20 or below. To reduce variability in the depth of sequencing between samples, each technical replicate was subsampled to a maximum of 2 million reads. Reads were classified using mothur's classify.seqs() function using the taxonomy and methods described in Mizrahi-Man et al. [51]. Classified reads were filtered by confidence score that ensured a maximum 5% false classification rate at each taxonomic level as determined previously (phylum = 65, class = 80, order = 60, family = 80, genus = 90) [51]. A table of counts for each taxonomic level was generated per replicate and was standardized for the total number of reads sequenced per replicate. See supplemental materials for summaries of reads per sample and rates of classification (Table S1). All sequencing data are currently being submitted to dbGaP.

### Data analysis

Spearman rank correlation was calculated using cor in R [52]. Replicate data were combined for all following analyses. Principle components analysis (PCA) was done using prcomp in R [52] after quantile normalizing each individual to a vector of the mean abundances of each taxa using the preprocessCore library from bioconductor [53] and log10 transforming the data. Clustering significance was assessed using two methods. First, we considered the difference in Euclidean distances along PC1 and PC2 between pairs of data points from the same season or between seasons. In addition, a nearest neighbor test was performed as described by de Silva et. al, using k = 3 nearest neighbors [32]. Abundance differences between seasons were tested using four procedures: 1. using a modified metastats procedure (a paired t-test was used rather than a t-test to account for study design) with 5000 permutations [33], 2. a modified metastats procedure (a paired Wilcoxon rank sum test used rather than a t-test) with 5000 permutations, 3. metagenomeSeq differential abundance tests using subsampled reads [34], and 4. metagenomeSeq differential abundance tests using all reads per sample. A false discovery rate of 5% was applied to correct for multiple tests [54]. Diversity metrics were calculated using the vegan package in R [55] and significance assessed via paired t-tests (for seasonal comparisons) and t-tests (for sex comparisons).

The Hutterites are a founder population with some degree of relatedness between all individuals in the population. Moreover, families and partners that reside in the same household are used in these analyses. We do not correct for relatedness in these analyses for two reasons. First, relatedness remains constant between seasons and therefore should not be a confounding variable for any cross-seasonal test with a paired design. Second, we examined pairwise Spearman correlation of bacterial abundance across all taxonomic levels among individuals living separately and living together (who are married, parent-offspring, or siblings) and did not find evidence for higher correlation of data from the individuals who cohabitate or are related (Figure S2).

### Questionnaire analysis

Seasonal differences in the consumption of 61 food categories were assessed (using a t-test) by averaging the quantities reported over 7 days for each individual. In addition, we combined categories (example: “produce”, “carbohydrates”, “dairy”, etc) to examine broad food groups. P-values were adjusted using the Benjamin Hochberg method to account for multiple tests.

### Enterotype analysis

Enterotype analysis was done using the standardized genus counts for all of the samples (replicates combined) as performed by Wu et al. [12]. Definitive enterotypes were not apparent, given that silhouette scores were low (<0.5) and cluster boundaries were not consistent when samples were processed separately by season and when both seasons were processed simultaneously.

### Yatsunenko et al. data comparison

V4 16S rRNA data from Yatsunenko et al. [2], which were generated using the same library preparation and sequencing protocol used in this paper, were downloaded from MG-RAST (http://metagenomics.anl.gov/) on 12/14/2012. Fasta files were run through our bioinformatics pipeline described in the data analysis section above to ensure consistent data processing between both sample sources.

### Metagenome prediction using PICRUSt

Closed reference OTU picking was performed using the subsampled reads against a Green Genes reference taxonomy (ftp://greengenes.microbio.me/greengenes_release/gg_13_5/gg_13_5_otus.tar.gz, downloaded 12/18/13) using the pick_closed_reference_otus.py script in QIIME [56]. 16S copy number was normalized using the normalize_by_copy_number.py script, metagenome functions were predicted using the predict_metagenomes.py, and data summarized into KEGG pathways using the categorize_by_function.py script, all included in PICRUSt [45]. Statistical comparisons were made using a paired Wilcoxon rank sum test and multiple tests were controlled using q-values.

## Supporting Information

Figure S1 **Rarefaction curves at each taxonomic level.** Rarefaction curves demonstrate that by sampling up to 2 million reads per replicate we detect most common and rare taxa, across the 5 taxonomic levels classified (generated using the R package vegan v2.0–3). In addition, evidence that winter has higher richness is clear in these plots, as in general the winter samples have a higher number of detected taxa than summer.
(EPS)

Figure S2 **Distributions of pairwise correlations by household.** A) Across all taxonomic levels examined, individuals living in the same household are not significantly more similar to each other (pairwise Spearman correlation) than individuals living in different households. B) Related individuals are not significantly more correlated in terms of microbiome composition than individuals residing in different households. Key: “separate” refers to unrelated individuals living in separate households, “siblings” refers to siblings living within the same household, “parent/child” are parent/child relationships of individuals who live in the same household, “married” are unrelated individuals who are married and live within the same household.
(EPS)

Figure S3 **“Full” dataset principle components analysis.** Principle components analysis (PCA) of all individuals collected in either season who were not on antibiotics 6 months prior to sampling (winter (blue) n = 94, summer (red) n = 92). Similar clustering by season is observed as with the n = 60 individuals that were sampled in both seasons (see Figure 2).
(EPS)

Figure S4 **Comparisons of differential abundance methods.** Each plot shows along the x-axis a q-value cutoff and along the y-axis the number of bacterial taxa that were called significant at that cutoff. For each plot the number of bacteria that were called differentially abundant in both tests is indicated by the darkest gray color, the number of bacteria that were not called differentially abundant by both tests are in the lightest gray color, and the bacteria that were called significant in one test but not the other are indicated by the medium shades of gray. In general, the metagenomeSeq analyses yielded more differentially abundant taxa than the metastats tests. A) Comparing metagenomeSeq analysis on subsampled sequences to metagenomeSeq analysis on all reads. B) Comparing metastats with a paired t-test to metastats with a paired Wilcoxon rank sum test. C) Comparing metastats with a paired t-test to metagenomeSeq analysis on subsampled reads. D) Comparing metastats with a paired t-test to metagenomeSeq analysis on all reads. E) Comparing metastats with a paired Wilcoxon rank sum test to metagenomeSeq analysis using subsampled reads. F) Comparing metastats with a paired Wilcoxon rank sum test to metagenomeSeq analysis using all reads.
(EPS)

Figure S5 **Enterotype analysis across all 120 samples.** A) Enterotype analysis using data from winter samples. B) Enterotype analysis using data from summer samples. C) Enterotype analysis using data from both winter and summer samples. For each A), B), and C), Left) average silhouette width for the best number of estimated clusters (3 or 4) is low; namely, there is little support for the three clusters as determined by k-means clustering. Right) Principle components analysis of Jensen-Shannon distance of genus level measurements, colored by enterotype assignment for each individual. Depending on how data are analyzed (whether seasons are analyzed separately or together), a different number of clusters are detected. D) The relative abundance of the dominant bacteria driving each enterotype classification in the Hutterite samples when both seasons are analyzed together. E) The three bacteria said to drive enterotypes in the orginal paper by Argumungam et al. do not distinguish enterotypes in the Hutterites. Gradients of both Bacteroides (gray) and Prevotella (yellow) are observed in summer (F) and winter (G).
(EPS)

Figure S6 **Diversity between seasons, sexes, and age.** A) A significant difference in Shannon diversity (H - left), richness (S - middle), and evenness (J - right) is observed between seasons (x-axis). B) H (left), S (middle), and J (right) between sexes is not significantly different in either winter or summer. C) Diversity decreases with age based on the data collected from winter samples (H (left): r2 = 0.116, P<0.01; S (middle): r2 = −0.0003, P = 0.33; J (right): r2 = 0.1068, P<0.01), but not based on the data collected from the summer samples (H (left): r2 = −0.0004, P = 0.33; S (middle): r2 = −0.007, P = 0.45; J (right): r2 = 0.003, P = 0.28). * = P<0.05, ** = P<0.01, *** = P<0.001, and **** = P<0.0001.
(EPS)

Figure S7 **Produce consumption varies by season.** In summer, when fruits and vegetables are grown in colony gardens, consumption of fresh produce is higher than in winter across the “full” dataset (t-test across seasons). Bars indicate standard error measurements. **** = P<0.0001, adjusted for multiple tests using Benjamin Hochberg correction.
(EPS)

Figure S8 **Abundance of predicted Glycan Biosynthesis and Metabolism enzymes varies by season.** In summer, abundance of predicted metagenome enzymes categorized as part of the Glycan Biosynthesis and Metabolism KEGG pathway are significantly more abundant than in winter (paired Wilcoxon rank sum test *P = *1.08×10^−6^).
(EPS)

Figure S9 **Comparison of data from the Hutterites with American, Amerindian, and Malawi gut microbiomes.** A) Principle components analysis (PCA) of the four populations (Hutterites colored by season of sampling). The distribution of the USA, Amerindian, and Malawi samples are similar to the original Yatsunenko paper (USA clustering separately from Amerindian and Malawi individuals). The Hutterites cluster separately from the other populations, however, given that different labs processed these samples it is unclear whether this separation is due to technical or biological sources of variation. The spread of the summer and winter Hutterite samples is smaller than the between population spread, demonstrating that the seasonal change in diet in the Hutterites is either smaller than diet or other influences on the microbiome in the Yatsunenko et al. populations. B) Diversity metrics (richness, Shannon diversity, and evenness) between populations. C) Diversity metrics (richness, Shannon diversity, and evenness) by age for each population. The Hutterites appear to have opposite trends for Shannon diversity and evenness as the Yatsunenko populations.
(EPS)

Figure S10 **Experimental design.** A total of n = 92 individuals in summer and n = 94 individuals in winter were sampled who did not take antibiotics six months prior to sampling. A total of n = 60 individuals were sampled in both seasons. DNA was extracted and libraries prepared in duplicate for each sample. Multiplexed libraries were sequenced across multiple flow cells, and sequences were processed using the bioinformatic pipeline described (see methods).
(EPS)

Table S1 **Sequencing summary by technical replicate and individual.** Sequenced: total reads from all lanes sequenced per sample. Pass_QC: number of reads that are maintained after the read QC procedure (see methods). Subsampled: number of reads after randomly subsampling to a maximum 2 million reads per technical replicate (maximum 4 million reads per individual per season). Classified at level: indicates the number reads that were successfully classified at each taxonomic level ensuring no more than a 5% false classification rate.
(XLS)

Table S2 **Paired metastats results (paired t-test).** This table contains raw P values for each taxa tested (by paired-metastats test, see methods), q-value, and average winter and summer abundances of each taxa tested.
(XLSX)

Table S3 **Paired metastats results (paired Wilcoxon rank sum test).** This table contains raw P values for each taxa tested (by paired-metastats test, see methods), q-value, and average winter and summer abundances of each taxa tested.
(XLSX)

Table S4 **Subsampled metagenomeSeq results.** This table contains for each taxa: the intercept, coefficient, and normalization factors from metagenomeSeq as well as the raw p-value, adjusted p-value, and q-value. Tests were run on the same subsampled reads (2 million per technical replicate) as the metastats tests.
(XLSX)

Table S5 **MetagenomeSeq results (all reads).** This table contains for each taxa: the intercept, coefficient, and normalization factors from metagenomeSeq as well as the raw p-value, adjusted p-value, and q-value. Tests were run using all reads generated and including library size as a covariate in metagenomeSeq.
(XLSX)

Table S6 **Dietary questionnaire results.** This table contains raw P values (t-test), average winter and summer daily servings, and Benjamin Hochberg adjusted P value for each food category (or groups of categories) tested for both surveys that overlapped the n = 60 individuals that were sampled in both seasons (surveys collected winter n = 31, summer n = 28) and the “full” dataset (surveys collected winter n = 41, summer = 45). Individuals were asked to specify how many times they ate each of 61 individual food categories (full question). These categories were combined into broad categories as indicated (subcategories) for certain comparisons.
(XLSX)

Table S7 **Differential abundance results of predicted metagenome KEGG categories.** This table contains raw P values (from paired Wilcoxon rank sum test), the mean winter and summer abundances, and q-values for each predicted KEGG pathway category tested.
(XLSX)
